# Draft Genome Sequences of 284 Xanthomonas citri pv. citri Strains Causing Asiatic Citrus Canker

**DOI:** 10.1128/MRA.01024-20

**Published:** 2021-01-07

**Authors:** Damien Richard, Adrien Rieux, Pierre Lefeuvre, Azali Hamza, Kanta Kumar Lobin, Mark Naiken, Randy Stravens, Claudine Boyer, Karine Boyer, Stéphanie Javegny, Olivier Pruvost

**Affiliations:** a Centre de Coopération Internationale en Recherche Agronomique pour le Développement (CIRAD), UMR PVBMT, St. Pierre, Réunion, France; b Agence Nationale de Sécurité Sanitaire de l'Alimentation, de l'Environnement et du Travail (ANSES), Plant Health Laboratory, St. Pierre, Réunion, France; c Université de la Réunion, UMR PVBMT, St. Denis, Réunion, France; d Institut National de Recherche pour l'Agriculture, la Pêche et l'Environnement (INRAPE), Moroni, Union of the Comoros; e Food and Agricultural Research & Extension Institute (FAREI), Le Réduit, Mauritius; f National Biosecurity Agency (NBA), Mahé, Seychelles; University of Arizona

## Abstract

High-quality Illumina assemblies were produced from 284 Xanthomonas citri pv. citri pathotype A strains mostly originating from the Southwest Indian Ocean region, a subset of which was also sequenced using MinION technology. Some strains hosted chromosomally encoded transcription activator-like effector (TALE) genes, an atypical feature for this bacterium.

## ANNOUNCEMENT

Most citrus-producing areas currently face Asiatic citrus canker due to the dissemination of its causative agent, Xanthomonas citri pv. citri pathotype A (*Xcc*A), from its native area (Asia) (1). This pathotype infects most cultivated citrus species. Based on minisatellite data, a single genetic lineage was found to be mostly responsible for the worldwide expansion of *Xcc*A (2). To better reconstruct the expansion of *Xcc*A and understand the genetic basis of its evolution, we sequenced the genome of 284 strains. Our sampling had a special focus on the Southwest Indian Ocean (SWIO) area (210 strains out of 284). The SWIO region comprises several geographically distant and administratively distinct islands, representing a good setting to apprehend *Xcc*A genetic structure and accessory gene content. In this region, Asiatic citrus canker management is attempted through integrated strategies, including using copper-based pesticides. Copper resistance involving a conjugative plasmid hosting the *copLAB* system recently emerged in Réunion, an outermost EU territory in the SWIO region (3). The sequenced strains were isolated from diseased citrus tissue using standard procedures (4) or were obtained from international culture collections, indicated by CFBP (Collection Française de Bactéries Associées aux Plantes, Beaucouzé, France) or NCPPB (National Collection of Plant Pathogenic Bacteria, York, UK) numbers. Copper-resistant strains (phenotypes were assessed as in reference 5) originating from all three regions of the world where this phenotype was reported were included. A further 60 *Xcc*A strains were selected from 16 countries to capture the global diversity of *Xcc*A. Genomic DNA (gDNA) was extracted from bacterial colonies grown on yeast extract-peptone-glucose agar (YPGA) medium using the Promega Wizard kit. Illumina sequencing was performed by GATC Biotech on a HiSeq 4000 instrument (2 × 150-bp format) using the genomic Nextera XT protocol. Long-read Oxford Nanopore MinION sequencing was additionally performed on 13 selected strains from gDNA purified using the SQK-RBK004 library preparation kit on a single R9.4.1 flow cell using the manufacturer’s instructions. Default parameters were used for all software unless otherwise specified. The Illumina reads were trimmed based on the quality score using Trimmomatic v. 0.36 with the options LEADING:15, TRAILING:15, SLIDINGWINDOW:4:20, and MINLEN:100 (6). *De novo* genome assemblies were generated using SPAdes v. 3.6.2 (7) for strains solely sequenced using short-read technology and Canu v. 1.8 (8), specifying a genome size of 5.3 Mbp, followed by Pilon v. 1.22 (9) for strains additionally sequenced using long-read technology. The genome size, number of contigs, GC content, and other metrics are detailed in Dataverse (10). Automatic annotation was performed using the NCBI Prokaryotic Genome Annotation Pipeline (11).

All the strains sequenced herein contained contigs similar to plasmids previously described in *Xcc*A. Accordingly, plasmids were identified in all strains submitted to long-read sequencing, most of them (ca. 60%) suggested as being circular by Canu. BLASTN searches identified the previously described copper resistance plasmid only in strains from Réunion but not in those collected from other SWIO islands.

In *Xcc*A, transcription activator-like effector (TALE) genes, including *pthA4*, which is necessary for disease development, and other so-called minor TALE genes are primarily plasmid borne (12). The sole exception was reported recently for pathotype A^w^ strains (with a host range restricted to Mexican lime and alemow) from Texas (13). Herein, 7 out of 13 long-read-based *de novo* assemblies contained minor chromosomal TALE genes, identified using Prodigal (14) and BLASTN. Whether these reads represent stable chromosomal integrations is presently being investigated further.

### Data availability.

The raw data are available under SRA accession numbers SRR11234551 to SRR11234624 and SRR11234626 to SRR11234855 and the corresponding BioProject information under accession number PRJNA599566. Details for each strain and the associated data accession numbers are available on the Dataverse platform (https://dataverse.cirad.fr/dataset.xhtml?persistentId=doi:10.18167/DVN1/TOVWWL) (10).
